# Implementing climate-sensitive health counselling: a qualitative study with physicians in Germany

**DOI:** 10.1186/s12913-025-13701-w

**Published:** 2025-12-01

**Authors:** Alina Herrmann, Silvan Griesel, Claudia Quitmann

**Affiliations:** 1 Heidelberg Institute of Global Health (HIGH), Faculty of Medicine, Heidelberg University, and University Hospital Heidelberg, Heidelberg, Germany; 2 Institute of General Medicine, Faculty of Medicine, Cologne University, and University Hospital Cologne, Cologne, Germany

**Keywords:** Climate change, Planetary health, Sustainable health care, Climate-sensitive health counselling, Patient-centered communication

## Abstract

**Background:**

To protect human health, it is important to improve climate-resilience and mitigate climate change and environmental degradation. Health systems can contribute to this by providing climate-resilient and sustainable health services. One example of such a health service is climate-sensitive health counselling (CSHC), which is defined as communication with patients about climate change and health issues within clinical settings. In this study, we explore how physicians in Germany conduct CSHC and what supports them in doing so with the purpose to better conceptualize CSHC and give recommendations on how to support its implementation.

**Methods:**

We conducted semi-structured qualitative interviews in 2021 with 18 German physicians mainly working in the outpatient sector, who were already conducting CSHC or thought about doing so. The interviews were audio-recorded and transcribed verbatim. A mixed deductive and inductive approach of qualitative content analysis was used for the analysis, supported by NVivo software. The deductive analysis part was guided by the framework on CSHC, and by work on factors that influence the implementation of patient-centered communication.

**Results:**

The way in which participants conducted CSHC was broadly aligned with the existing framework for CSHC. With regard to the frameworks’ three content areas (’health impacts and adaptation’, ‘healthy and sustainable lifestyles’ and ‘climate action and policy’), there was an emphasis on lifestyle counselling in this sample. Furthermore, a fourth content area on clinical-decision making was discussed. The most important communication strategies mentioned fitted to principles of patient-centered communication. Enabling factors for implementing CSHC were mentioned on all pre-defined levels (physician, patient, relationship and health system factors). Developing an inner attitude, positive experienced patient response and being aware of touchpoints for CSHC were mentioned as physician factors supporting implementation of CSHC by participants.

**Conclusions:**

This study demonstrates that participating physicians already implement CSHC as a sustainable healthcare service. As physician factors seem to be important enabling factors for implementing CSHC, we argue that training physicians in medical curricula and continued medical education are important to support implementation of CSHC. Reflections about the own attitude towards climate change and health and teaching about patient-centered communication strategies could be important elements of such trainings. Future research should consider the expansion of the CSHC framework with regard to clinical decision-making and validate or expand the framework for different medical specializations.

**Supplementary information:**

The online version contains supplementary material available at 10.1186/s12913-025-13701-w.

## Background

Humanity is crossing planetary boundaries such as climate change [1], endangering its own livelihoods and health [2]. To protect human health and well-being, there is an urgent need for climate-resilience and decarbonization [3]. The German Advisory Council on Global Change (WBGU) states that “harnessing the transformative potential of health systems” is key to achieve “healthy living on a healthy planet” [4]. Climate-resilient and sustainable health systems can help to protect health from such effects and at the same time minimize their environmental impact [5]. Moreover, health professionals can address climate change as a health issue and promote healthy and sustainable societies as a whole [6, 7].

Therefore, a promising health care intervention to improve climate-resilience and sustainability is climate-sensitive health counselling (CSHC) [7–9]. CSHC means communicating with patients about climate change and health issues in clinical settings [8]. A scoping review has identified three aims of CSHC: to protect and promote individual and public health, to increase climate change and health knowledge and awareness and to encourage climate action and lifestyle change [8]. So far, only few studies have examined the actual effects of CSHC [8]: It has been demonstrated that CSHC can contribute to raising awareness and improving knowledge of climate change and health [10, 11]. However, it remains unclear whether other aims, such as promoting health or encouraging climate action, can be reached.

Research has largely investigated health professionals’ knowledge on climate change and health and their willingness to discuss related issues with patients [12–16]. A recent review suggests that health professionals are increasingly aware of the link between climate change and health, and are willing to talk about it with patients [17]. In a non-representative survey of 1,683 German physicians conducted in 2021, 83% said they would be willing to conduct CSHC [15]. While we are not aware of any representative studies about its implementation rate, in a population-based panel study across five federal states in Germany in 2022, 8.7% of patients reported to have received CSHC before. This suggests that CSHC is not widely implemented yet. However, there is an increasing interest for CSHC across Germany, as it is being promoted by some physician networks [18], non-governmental organisations [19], institutions for continued medical education and within specific and localized health care models of statutory health insurance [20].

Reasons for lacking implementation have been identified. For instance, Redvers et al. (2024) interviewed Canadian physicians about patient-planetary health co-benefit prescribing, a concept close to CSHC. They found that the current healthcare system does not support planetary health and that clinicians find it difficult to incorporate it due to cultural barriers and missing capacities [21]. Other studies have found that a lack of knowledge and skills, time constraints, and the feeling that climate change is too political or that CSHC will not make a difference, hinder health professionals from implementing CSHC [8, 17].

An increasing number of studies investigate how to overcome such barriers and contribute to the conceptualisation of CSHC [7, 17]. Griesel et al. explored what factors impact patients’ acceptance of CSHC and found that emphasizing the link of climate change to individual health, patient-centred communication, a good therapeutic relationship and physicians’ credibility could be beneficial [22]. A recent experimental study showed that health-only framings of lifestyle counselling were more acceptable than climate-and-health framings [23]. Den Boer et al. explored US physicians’ perspectives on CSHC and found that they were positive about it, especially, if CSHC could help to protect their patients’ health. The authors stressed that further studies need to examine how such counselling could be facilitated, as physicians in their study had not yet been conducting CSHC [24]. Similarly, Campbell et al. concluded that future research needs to investigate how programs for CSHC need to be designed to support implementation of CSHC [17].

Therefore, we explore how physicians in Germany conduct CSHC (research question 1) and what supports them in doing so (research question 2) with the purpose to better conceptualize CSHC and give recommendations on how to support implementation of CSHC, respectively.

## Methods

### Research design

In this cross-sectional qualitative study, we conducted semi-structured qualitative expert interviews [25] with physicians in Germany. In this method, an expert is someone who is responsible for designing or implementing an activity under study, and who has privileged access to information about the phenomenon of interest [25]. The study was part of a bigger project conceptualizing CSHC. Further sub-studies of this project included a literature review [8] and quantitative and qualitative insights in patients’ perspectives on CSHC [22, 23, 26]. No framework on CSHC existed at the conception of the study and little was known about the phenomenon overall [8], so that no theoretical framework on CSHC was available to guide the conception with regard to research question 1. With regard to research question 2, the authors had originally planned to use implementation frameworks [27, 28], they had used before [29] to identify factors supporting implementation of CSHC. We applied the COREQ-guideline on reporting qualitative research to present methods and findings of this study [30].

### Sampling and recruitment

We applied a purposeful sampling strategy by selecting information-rich cases, which allowed us to investigate the phenomenon under study in-depth [31]. With respect to our research questions, information-rich cases were physicians who already had conducted or thought about conducting CSHC. We also included the option of “having thought about conducting CSHC” into our inclusion criteria, because we wanted to include physicians who might not feel confident enough to say that they were already conducting CSHC. At the time of data collection, first talks were given about CSHC (named “Klimasprechstunde”/Climate consultation hour [32, 33]) in the climate change and health community in Germany, so that we expected health professionals to consider conducting CSHC, but not having much experience. Recruitment took place via mailing lists of the German Alliance on Climate Change and Health (KLUG e.V.) and the section on Climate Change and Health of the German College of General Practitioners and Family Doctors (DEGAM). All individuals responding to the initial e-mail and fulfilling the inclusion criteria (health professionals who already had conducted or thought about conducting CSHC) were interviewed. Initial interviews also including other health professions than physicians (dietician, physiotherapist, pharmacist, medical student) revealed great divergence from approaches by physicians, which would not have allowed us to reach data saturation given the limited capacities of this research project. We therefore decided to focus the recruitment on physicians. In post-interview debriefings AH and SG sought for data saturation. In particular, we discussed whether any new topics emerged, ensuring that the dataset was comprehensive with regard to the research questions and that there was a great enough amount of repetition to identify commonalities between individual participants’ statements [34].

### Data collection

The interview guide (Appendix 1) was developed based on theoretical guidance by Gläser and Laudel (2010) [35] and guided by the research questions. It used open-ended questions covering the following main topics in relation to CSHC: previous experiences, aims, topics, target groups, communication strategies, touchpoints, skills and attitudes. As there was very little literature about CSHC at the time, the interview guide was constructed exploratively without prior frameworks. It was pilot-tested with two physicians, resulting in minor changes of ordering and formulation of questions. At the beginning of each interview, information was collected on the following: age, gender, medical specialization, work environment (single or joint practice, hospital), and the federal state in which the participants worked. From February to April 2021, interviews were conducted in German by AH alone (a medical doctor and experienced qualitative researcher) or AH and SG (a medical student at the time). Some of the interviewees were known to AH through her professional network. All interviewees were informed about the objectives of the research. Due to Covid-19 restrictions, all interviews were conducted via video call, which is a suitable alternative to in-person interviews [36].

### Data analysis

Interviews were audio-recorded and transcribed verbatim. We analyzed the data with a mixed deductive and inductive approach of qualitative content analysis [37], supported by NVivo Software (Version 14) and field notes taken during the data collection. The content-structuring content analysis (“*inhaltlich-strukturierende Inhaltsanalyse*”) by Kuckartz is a qualitative analysis method, which helps to identify and thematically structure the central manifest content of a dataset [37]. In short, after a familiarization phase, main categories are developed mainly deductively guided by the research questions. Then, text material is sorted into the main categories and sub-categories are developed mainly inductively. Finally, the full text material is coded following the final coding scheme [37].

While the interviews were conducted in 2021 when little was known about CSHC, the main analysis was conducted in 2024. By this time, the research team had expanded its knowledge of CSHC through other sub-studies of the wider project on conceptualizing CSHC, and more literature was available overall. As the first research question “How do physicians conduct CSHC?” was always directed to contribute to the conceptualization of CSHC, the analysis was based on the CSHC framework, which was developed by the research team itself, describing the ‘aims’, ‘content areas’ and ‘communication strategies’ of CSHC as well as the guiding principle of ‘integration into routine care’ [8]. The results section will present in more detail where the data was consistent with the original framework or exceeded it, leading to inductively added codes.

For the second research question, about what supports physicians to implement CSHC (RQ2) the deductive-inductive coding process was less straightforward. While the research team had considered to use a common implementation framework [27, 28] to describe factors supporting implementation of CSHC, sub-studies within the wider project on conceptualizing CSHC had already identified patient-centered communication as a potentially important principle for CSHC [8, 22] and it emerged as an important principle in familiarization phase of this dataset. In an iterative process of initial inductive coding, team discussion and consultation of the literature, we finally decided to draw on work from Epstein et al. (2005), which sorts factors influencing the implementation of patient-centered communication into four categories (patient factors, clinician factors, relationship factors and health system factors) [38]. Those categories are still similar to other implementation frameworks [27, 28], but differ mainly by having a ‘relationship’ category, which resonated well with the findings in our dataset. We used the four categories by Epstein et al. [38] to build the deductive coding scheme for our main categories of the analysis of RQ 2. Sub-categories were mostly developed inductively although there was some overlap with sub-factors mentioned in the work from Epstein et al. (2005).

The full coding scheme was discussed during regular meetings (CQ, AH) and validated by SG in two final meetings. AH and CQ were both involved in the coding process. The final framework is presented in the Appendix 2 and 3, including detailed information which categories and codes were applied deductively and which ones emerged inductively. Transcripts and results were not shared with study participants for feedback. Quotes presented in this manuscript were translated with support of an artificial intelligence-based translation tool (deepL.com) and revised by the authors and a bilingual colleague.

### Research team and reflexivity

AH, SG and CQ are members of KLUG e.V. and AH is spokesperson of the DEGAM section on Climate Change and Health. The research was inspired by the discourse on CSHC within these organizations and the lack of research on the topic at the time. Our own attitude towards climate change and health and the membership in these organizations was critically reflected throughout the research process, guided by the idea, that the undertaken research should be relevant for the whole health care sector and not the narrow climate change and health community only.

### Ethical considerations

The study protocol was approved by the ethical board of the medical faculty of Heidelberg University (S-917/2020). Participants were informed about the study, including the recording of the interview. Written informed consent was obtained from all participants.

## Results

### Sample description

The final sample consisted of 18 physicians, slightly female-dominated with an average age of 51 years. Most participants were general practitioners (*n* = 11) and worked in out-patient care (*n* = 16) (see Table 1). The workplaces were in 10 out of 16 German federal states, including smaller and bigger cities as well as rural areas. On average, the interviews lasted 55 minutes and all physicians reported some concrete experiences with conducting CSHC.

**Table 1:** Sociodemographic data of the participants (*N* = 18)

Characteristics		N (%)
Age	30–39	5 (28%)
	40–49	4 (22%)
	50–59	6 (33%)
	> 60	3 (17%)
Gender	Male	6 (33%)
	Female	12 (67%)
	Non-binary	0
Medical specialization	General and family medicine	11 (62%)
	Internal medicine	4 (20%)
	Gynecology	1 (6%)
	Orthopedics	1 (6%)
	Neurology and psychiatry	1 (6%)
Work environment	Out-patient care	16 (89%)
	In-patient care (hospital)	2 (11%)

### Research question 1: how do physicians conduct CSHC?

Guided by the framework of CSHC [8], we used four categories to describe how physicians conducted CSHC: Aims pursued by physicians, addressed content areas, applied communication strategies, and practiced integration into routine care.

The interview questions focused on climate change, and specific questions about planetary health were asked at the end. Participants partly referred to planetary health and other environmental issues, such as biodiversity, air quality, animal welfare and sustainability throughout all of the interview.

**Fig. 1:**
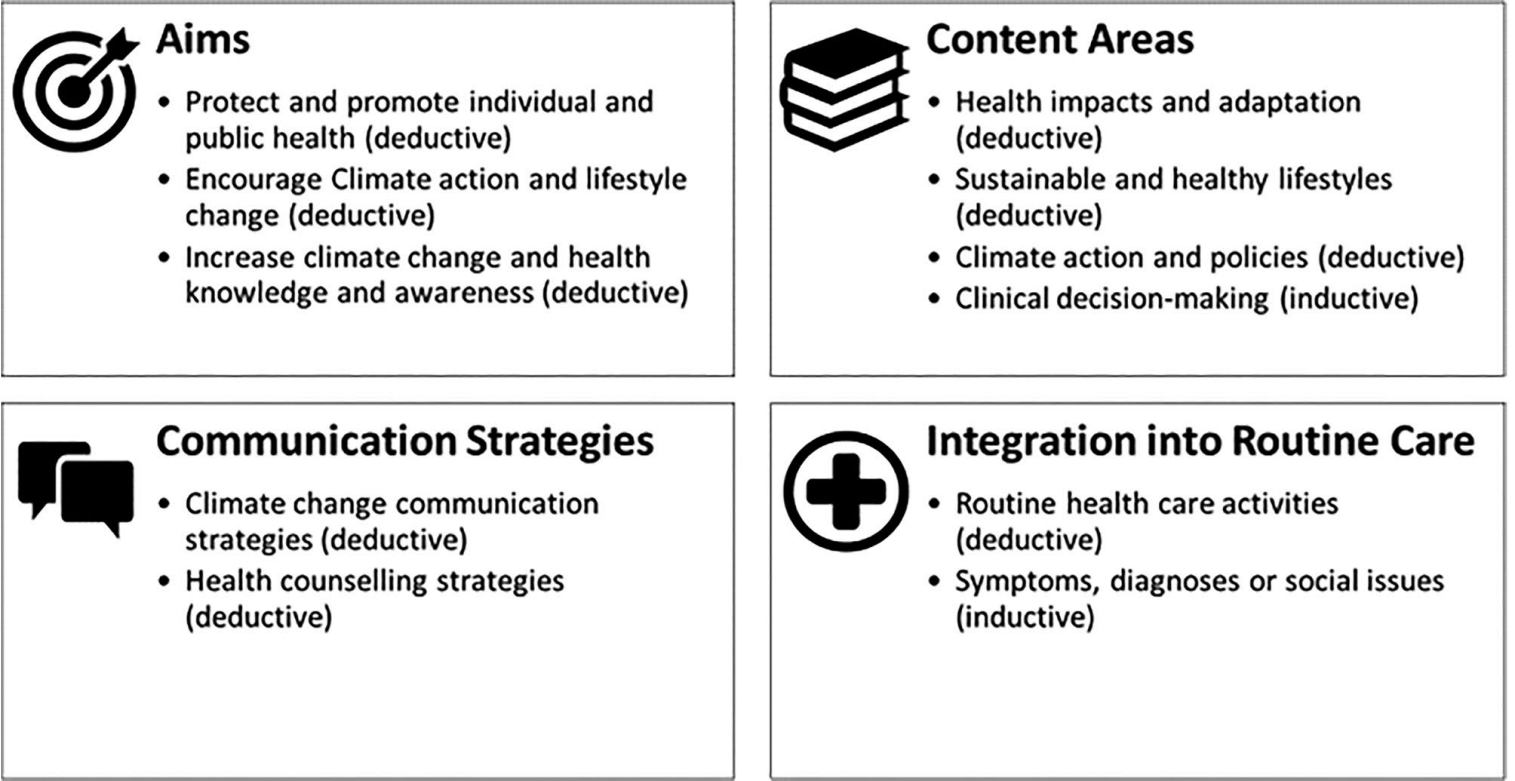
Main and sub-categories of coding scheme for research question 1. The four main categories were derived deductively from the framework of CSHC [8]. Sub-categories were either derived deductively from the framework or inductively, as indicated in parenthesis

The main and sub-categories of the coding scheme for research question 1 are presented in Fig. 1. Appendix 2 shows the coding scheme including all codes.

#### Aims

Three main aims were identified, in line with the preexisting CSHC framework. First, physicians wanted to protect their patients’ individual and public health. Second, most physicians wanted to promote climate action and lifestyle change. Some physicians not only wanted to encourage patients’ lifestyle change in terms of their “carbon footprint”, but also climate action on a broader societal scale:

Interviewer: “What aims do you pursue when addressing climate change in doctor-patient consultations?” – “In the short term, of course, the health of the patient sitting in front of me. To make her realise that she can do a lot for herself through her behaviour. But I have to say, I’m also pursuing a bit of a political aim. Of course, I hope that by addressing this, I’m also sending out a signal that these are issues, where we are collectively threatened. […] We also want to go beyond purely individual behaviour and say: Okay, we can do this together. (P14, female)

In this regard, many physicians mentioned that they wanted to strengthen patients’ self-efficacy and the belief, that they could make a difference for themselves and the planet:

And that’s when the patients get involved, when they simply realise: Okay, if I eat differently, if I go into the forest, if I plant trees, if I fly less and so on, I can make a contribution. (P11, male)

Third, many physicians felt that raising knowledge and awareness of patients was an important aim. While some physicians explicitly wanted to raise knowledge and awareness about the nexus between climate change and heath, other topics were the climate crisis in general, climate justice, air pollution or being part of nature.

#### Addressed content areas

One important part of the CSHC framework [8] are the “content areas”: topics that can be addressed in CSHC. In this study physicians touched upon all three content areas of the framework (‘health impacts and adaptation’, ‘sustainable and healthy lifestyles’ and ‘climate action and policies’) and brought up a fourth one covering ‘clinical decision making’.

##### Health impacts and adaptation

Heat was the most commonly mentioned climate-sensitive phenomenon relevant to health, and often participants elaborated on adaptation advice given to patients. Physicians also named other phenomena and diseases, that they addressed with patients, such as mental health, vector-borne diseases, pandemics, allergies, air pollution, UV-radiation, and biodiversity loss.

##### Sustainable and healthy lifestyles

Many participants focused on sustainable and healthy lifestyles. Concerning dietary behavior, physicians talked about reducing meat, dairy products and ready-made meals, increasing dietary diversity and eating a more plant-based diet. Some explicitly said that they recommended the ‘10 rules of the German Nutrition Society’ or the ‘Planetary Health Diet’ to their patients. Regarding mobility behavior, most physicians addressed that they tried to *“motivate people to get into the habit of doing more everyday exercise, leaving the car at home more often and switching to other forms of transport.” (P18, female).* Using the bike was recommended to patients most often as active mode of transport. Some physicians reported that they assessed in which mode of transport patients commuted in the medical history to find a touchpoint for CSHC.

Several physicians also addressed the importance of connecting with nature, often combined with mindfulness aspects. Physicians reported advising patients that connecting with nature reduces stress, alleviates depressive symptoms, and increases well-being. Participants also shared underlying personal views about “*nature spaces as healing spaces*” (P1, male). Connectedness to nature was also seen as an opportunity to reduce emissions related to drug therapy (e.g. antidepressants) or long-distance travel: *“Then they [patients] realise, that we don’t have to travel so far to discover beautiful things.”* (P3, female)

A last lifestyle aspect brought up by physicians was unnecessary consumption or overconsumption of vacation, fashion, social media or food. Such (over-)consumption by patients was seen as defense mechanism or escape from conflicts, negative emotions or inner imbalance. One physician said that she wanted to convey patients that “*contentment and happiness either arise from being content with myself or from cultivating relationships with others. And that this has nothing to do with consumption.*” (P17, female)

##### Climate action and policies

Most physicians said that they discussed climate action and policies with their patients under certain circumstances. These conversations typically arose when patients expressed concern about climate change or experienced climate-related anxiety. In such cases, discussing climate action was seen as a way to support patients’ well-being by enhancing their sense of self-efficacy and fostering a feeling of solidarity:

‘I’m not alone, I don’t need to be afraid, but my concern is justified.’ Perhaps that’s how I would put it if I were talking to a patient with anxiety disorder. And what can I do to reduce my anxiety? Regaining my ability to act, by simply taking action myself, by organising myself, by doing something, by becoming a member of some association where I plant trees, by getting involved with Greenpeace etc. etc. etc. (P14, male)

Some physicians shared that they addressed broader political contexts to create a feeling of shared responsibility in society, for example by naming the responsibility of policy-makers to create adequate bike infrastructure to encourage active travel. Physicians stressed that they tried to remain politically neutral, for example by not naming political parties.

##### Clinical decision making

Another aspect, not being part of the CSHC framework, which was addressed by few participants was clinical decision making. Here, physicians talked about reducing overdiagnosis and overtherapy, switching to more environmentally friendly medication, such as dry powder inhalers instead of metered dose inhalers, or prescribing natural therapies such as phytotherapy or hydrotherapy. One physician reported that she had the environmental impact in mind, when she dissuaded patients from receiving unnecessary treatments or diagnostics, but did not mention that additional diagnostics were harming the climate, because she felt that many patients would already “*adopt a defensive stance*” (*P4, female*), if diagnostics were denied.

#### Applied communication strategies

We identified strategies primarily known from climate change communication and from health counselling, as proposed in the CSHC framework.

##### Climate change communication strategies

With regard to climate change communication strategies, almost all physicians reported to use a co-benefit framing to align health with climate change mitigation: For instance, one participant said that his main message was that “*a climate-friendly life can also be fun, fulfilling, meaningful and good for your health.*” (P9, male). Most participants also reported that ‘being a role model and acting authentically’ was an important strategy:

Well, I live 34 kilometers away. But I always cycle and take the train […]. There are quite a few patients who see that I come by bike […]. And then I always have my bicycle helmet lying demonstratively behind me on the windowsill, (laughs) so that’s always a little springboard when they ask, ‘Oh, did you cycle to work? Where do you live?’ and then you sometimes get into a conversation about it. (P3, female)

##### Health counselling strategies

With regard to health counselling strategies, almost all strategies identified in the CSHC framework were mentioned, for instance: patient-centered communication, shared decision making, active listening, motivational interviewing, strength-based approach, and a narrative approach. Many physicians emphasized patient-centered communication, so that we described this sub-category (see Appendix 2) more in detail as a second level sub-category by using the definition of Epstein et al. [38] to define the four second level sub-categories. Table 2 provides codes and quotes from the four second level sub-categories of ‘patient-centered communication’ to give insides into how physicians implemented patient-centered communication in CSHC.

**Table 2:** Further illustration of the sub-category of ‘patient-centered communication’ in CSHC (see Fig. 1 for main categories)

Sub-category	**2**^**nd**^ **Level Sub-categories**	Codes	Exemplary quote
**Patient-centered Communication**	Eliciting patients’ perspectives	Interest in patient perspectives	So, curiosity and openness and wanting to understand the other person. These are important points for me, then relationships can develop and then a lot of change is possible. (P17, female)
		Exploring patients’ responsiveness to CSHC	If I see that this topic is not well received or even responded to with resistance, then I quickly withdraw from the discussion. (P12, female)
		Reacting on topics brought up by patients	The foresters actively bring their concerns to the consultation. Due to the climate, especially all the nature-related professions, they observe a lot. Or the concerns of patients about the heatwaves they experience. (P1, male)
	Considering biopsychosocial context	Not doing CSHC with all patients based on their personal situation	I also have severely impaired patients […] so they are not able to deal with the quasi ‘luxury problem’ of the climate crisis. So, on the Maslow pyramid of needs, they are, so to speak, on the very banal needs that have to be secured. And they’re really struggling, so they don’t have to answer the question: What meaningful things can I do? (P17, female)
		Respecting patients’ limited opportunities	For example, assembly workers, […] sometimes they just go somewhere for a whole week and then they say, ‘Yes, what am I supposed to do there?’ Someone then says, ‘I don’t have much money. Then we go to the supermarket and buy a quick meal for lunch and can’t cook for ourselves. And how are we supposed to influence that in any way?’. I find it very difficult to give them tips on how they should somehow change their diet to a higher standard. (P15, male)
		Adjusting aims and contents of CSHC to individual patient	The heat in summer, that was a huge issue. […] I always do that individually. When I realise that there’s a patient who is just with himself, then I just give advise how to deal with himself/But if I realise that he is also someone who is receptive to more, then I go so far as to say: okay, then you might have to help or support and influence the politicians. And that is always the highest goal that I then express. (P8, female)
	Reaching shared understanding	Looking for possibilities for change together	But I don’t try to say: ‘You have to do this and that’, but I try to work out with the patient where there are fields of action for them and where they can make small changes somewhere in order to set themselves up in a better way. (P12, female)
		Searching for resonance	The opportunity arises because someone brings up a certain topic. And then I notice how this person resonates with me, and I realise that I can expand on this again. (P2, female)
	Share power and responsibility	Making shared decisions	And I am also convinced that I only give advice and that the decision is always taken together. Because I simply believe that this is also respect for the other person. Respect for the other person’s life and for the difficulties that everyone has in their life, which I can’t judge at all. (P8, female)
		Giving patient responsibility for healing	Educating and motivating patients to basically be their own therapists. And by being that, in the naturopathic sense, they are also therapists for society and the planet.’ (P11, male)

A practical aspect, that was discussed throughout the category of patient-centered communication, was the question whether climate change should be mentioned explicitly. Some participants stated that they did not mention climate change explicitly or were unsure about doing so, because they feared negative patient reactions.

#### Strategies for integrating CSHC into routine care

Most physicians stressed that they integrated CSHC into routine care, looking for touchpoints for CSHC. In the CSHC framework the overarching principle is called “Integration into routine health care activites”. In this sample of physicians, participants thought of this integration from the perspective of ‘health care activities’, such as check-ups, discussion of results of medical examinations or regular lifestyle counseling and from the perspective of ‘symptoms, diagnoses, or social issues’, such as obesity, diabetes, cardiovascular diseases or mental health issues. Therefore, we slightly adapted the name of the deductive main category to “integration into routine health care”. Appendix 2 provides a detailed overview of the touchpoints mentioned.

### Research question 2: what supports physicians in conducting CSHC?

**Fig. 2:**
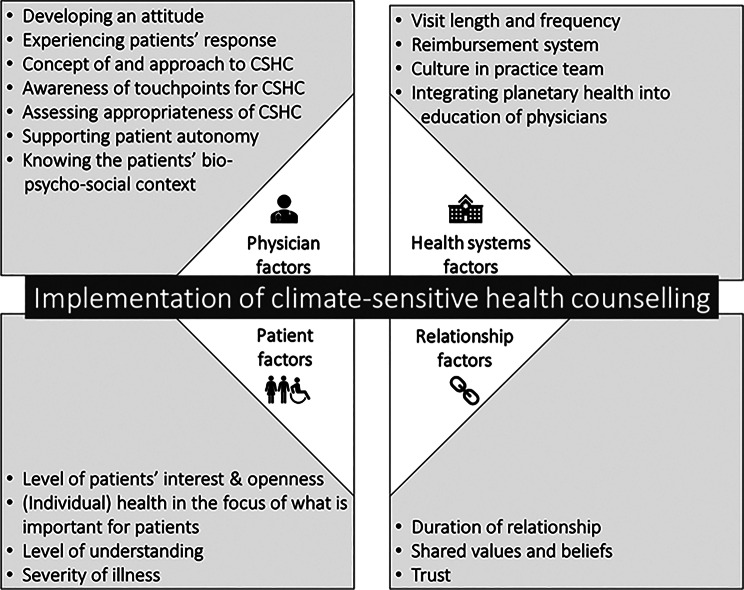
Factors influencing the implementation of climate-sensitive health counselling (CSHC); the four main categories were applied deductively based on existing work on factors influencing patient-centered communication [38]

Guided by work on factors influencing the implementation of patient-centered communication [38] we analyzed the results deductively along four categories: health systems factors, clinician factors (physician factors in our case), relationship factors, and patient factors, with physician factors being mentioned most often. Figure 2 summarizes the main and sub-categories, which influenced implementation of CSHS. The coding scheme is presented in Appendix 3.

#### Physician factors

The physician factors will be presented along the sub-categories. An excerpt of the coding scheme and quotes are presented in Table 3.

**Table 3:** Excerpt of the coding scheme for physician factors (full coding scheme in Appendix 2)

Sub-category	**2**^**nd**^ **level Sub-category**	One exemplary code	Exemplary quote
**Physician Factors**	Development of an inner attitude	Personal motivation regarding climate change and health	“The inner motivation is that the climate crisis is ultimately present and visible everywhere. And it’s perceptible, not just when you switch on the TV and watch films about the Arctic or Antarctic, but it’s apparent here on the ground and the changes are perceptible in hotter summers, drier forests, an increase in allergies, lung diseases, asthma and COPD. My counselling is also more along the lines of getting more vaccinations, for example, because TBE is spreading. And I have two small children and, of course, I want them to experience the world as it is for us, as we have come to know it and with an intact ecosystem and not with a very reduced biodiversity.“(P12, female)
	Experiencing patients’ response	Positive patient response	“And if the patient is interested, I either mention it in the next sentence that this is also about conserving resources and the climate and whether they have ever thought about it. But never in a directive way. Sometimes I am/I always work with the patient’s situation. So, I’m always very attentively interested. And that goes down really well with people. I was amazed myself at how many are already thinking about these questions.” (P1, male)
	Assessing appropriateness of CSHC	Reasons for appropriateness	“As a doctor, I feel like I am in charge of my patients’ health. I don’t necessarily feel responsible for the health of my patients, because of course they also bear a lot of responsibility themselves. But of course, I make a relevant contribution by prescribing medication, by talking to them, by giving them medical advice or something like that. And climate change also has a very direct impact on my patients. That’s why I think it’s absolutely my role to make sure that it doesn’t get worse. Because it also affects my individual patients. So, the circle is complete.” (P4, female)
	Concepts of and approach to CSHC	Own approach to CSHC	“No, I do that rather intuitively, spontaneously. So, if we’re talking about nutrition and someone says they’re going vegetarian or something like that, then I’ll say something or if it’s about lifestyle diseases, high blood pressure, obesity, lack of exercise and things like that. Then I say: ‘It’s much more fun and more active and it’s better for the environment and the animals and the climate or something, yeah.” (P9, male)
	Awareness of touchpoints for CSHC	Based on specific symptoms or diagnoses	“So for me, the umbrella term climate consultation means always considering the topic of climate and health in my regular consultations […] always having it in mind and looking for touchpoints with each patient anew and then talk about it, if it is suitable.” (P4, female)
	Supporting patient autonomy	Not being authoritarian, paternalistic or intrusive	„And so, I would never want to patronise the patient. I would never want to tell them what to do.” (P3, female)
	Knowing the patients’ bio-psycho-social context	Knowing the patient	*“If you look after patients for a long time, at some point you gain an insight into the family, into the generations of the family. Because it’s often family medicine. So there are several people from one family and you always have a personal chat with those you look after more closely. And over time, you get to know each other and you can clarify your attitude and position.”(P12, female)*

##### Development of an inner attitude

The personal factors of the physicians, who were pioneers in the field of CSHC, was prominently discussed by the participants. Participants had a high level of personal motivation regarding climate change and health, and sometimes also mentioned interest in other environmental issues, such as biodiversity. Many physicians reported a long personal history in the environmental movement or a key event which served as a starting point for their professional engagement with the topic of climate change and health. Some reported explicitly, that they tried to live in a climate-friendly way.

The participants emphasized that they perceived a responsibility to conduct CSHC, for example because of the urgency to mitigate climate change. Some described how conducting CSHC gave them a feeling of self-efficacy with regard to climate action. About half of the participants said they engaged in climate change and health activities beyond CSHC.

##### Experienced patients’ response

If physicians already had experienced patients’ responses, it rather supported them in continuing to do CSHC (see Table 3). Overall, positive patient response was reported. Some physicians reported that patients were surprised, either by information transmitted during CSHC, or by the fact that physicians were talking about climate change and health at all. Seldomly physicians reported that patients refused CSHC actively. Some said that they did not really know whether patients perceived CSHC positively or negatively. In terms of impact, several physicians reported not expecting too much impact. However, few reported the feeling of having an impact, mainly by reinforcing patients’ existing intentions or by providing them with food for thought.

##### Concepts of and approach to CSHC

Many participants raised the problem that there was no clear definition and concept of CSHC. Some added that they were in the process of finding their own approach to CSHC, which they felt took time. Some participants mentioned that they had listened to talks of other physicians conducting CSHC and had discussions with colleagues during journal clubs or workshops about climate change and health communication, which they found helpful in this process. Moreover, few participants referred to literature, for example, on climate change communication, citing it as a valuable source of guidance.

##### Awareness of touchpoints for CSHC

Many participants described being aware of multiple touchpoints, which could be used to integrate CSHC into routine health care activities (see also research question 1). Being aware of these touchpoints was seen as an enabler for integrating CSHC into daily work. One participant reported “to screen” (P8, female) every patient for a suitable touchpoint for CSHC during the consultation.

##### Assessing appropriateness of CSHC

Participants mostly felt CSHC to be appropriate due to the scientific evidence on the health impacts of climate change and other environmental crises, the potential of health professionals as multipliers, the fact that CSHC is about disease prevention, and the positioning of professional associations on climate change and health. Some aspects were seen to be inappropriate, for example certain (political) statements or if CSHC was not aligned with the interests or needs of patients.

##### Supporting patient autonomy

Supporting patient autonomy, rather than being authoritarian, paternalistic or intrusive, was emphasized by almost all participants as important in the implementation of CSHC.

##### Knowing the patient’s bio-psycho-social circumstances

Some physicians explained that it was important to know the patient, including their living circumstances, in order to identify suitable patients for CSHC and to be able to conduct CSHC.

#### Patient factors

Many different groups of patients with a varying level of interest and openness towards CSHC were identified. Among those showing a higher level of interest and openness, younger patients, highly educated patients and patients with children or grandchildren were mentioned. Few participants reported that patients actively addressed topics related to CSHC. It was repeatedly emphasised that CSHC was facilitated, if the patient’s primary health concern was a suitable touchpoint.

“So, I always find it easy when the patient is directly affected. So that means, for example, the relatives who come with the grandma - it’s always easy for me to address climate change, because everyone knows this problem with drinking, for example, somehow during heat waves, not drinking enough, then the grandma feels bad. This connection, they all know that.” (P4, female)

Furthermore, chronic conditions requiring long-term behavioral changes were rather seen as an opportunity for CSHC, as opposed to severe and acute conditions, which were not considered to be the right context. Speaking about climate change explicitly was done, if patients showed some level of interest or understanding for the topic and not considered if it seemed to be too far away from patients’ reality.

#### Health systems factors

The length and the frequency of visits were identified as significant factors in the implementation of CSHC.

The advantage we have as GPs is that we don’t just see them [patients] once. So, we can gently place our topics and come back to them when the opportunity arises. (P3, female)

A culture supporting CSHC in the health care facility was described as helpful for implementation. Some participants regarded reimbursement of CSHC as a pivotal aspect, because this would serve as a political signal for instance. Others stated that, reimbursement would not make a difference for implementation for them personally. Some wanted climate change and health issues to be included as a mandatory part of routine health check-ups. Few participants proposed to incorporate planetary health into medical curricula and training programs or to establish additional certificates (”Zusatzbezeichnung)”.

#### Relationship factors

A good doctor-patient relationship was seen as an important enabler for implementation of CSHC.

The nice thing is that as a doctor you simply have a total leap of faith. They know I don’t want to sell them anything or do anything out of some kind of, yes, self-interest. And yes, most of them are very open as a result. (P10, female)

In addition, shared values, beliefs and trust were identified as important factors facilitating the implementation of CSHC.

## Discussion

### Summary

In this purposeful sample of physicians conducting CSHC in Germany, the reported aims, content areas, communication strategies, and principles for integration into routine health care resonated well with the structure of the pre-existing CSHC framework [8]. Yet, we can derive several additional emphases and potential extensions to the framework, which add to the conceptualization of CSHC (RQ1). With regard to the aim of encouraging climate action and lifestyle change, important nuances were added: participating physicians aimed to increase patients’ self-efficacy and support the believe, that they can make a difference for themselves and the planet. Regarding the content areas, clinical decision-making arose as potential additional content area of the CSHC framework. Concerning the communication strategies, this study emphasizes the importance of patient-centered communication. With regard to the overarching principle of integrating CSHC into routine health care, contextual factors like symptoms, diagnoses and social issues came up as additional touchpoint for CSHC. In consideration of the factors supporting the implementation of CSHC (RQ 2), this study with highly engaged physicians, draws the attention to physician factors, such as developing an inner attitude which enables CSHS, being aware of touchpoints for CSHC and experiencing positive patients’ responses.

### Research question 1: how do physicians conduct CSHC?

The physicians’ experiences underscore the CSHC framework in an empirical way and add nuances to its categories [8]. Regarding the aims, all three possible aims of CSHC were mentioned. However, not all physicians pursued all aims to their full extent. For instance, some physicians talked to patients about how to change their lifestyles in a healthy and sustainable way. However, they did not explicitly address the climate aspect with their patients. This was also pointed out as a potential strategy to avoid expected negative patient reactions by US physicians [24]. This means that physicians convey practical knowledge supporting climate action and lifestyle change, but might not pursue the aim of raising awareness of climate change and health. As mentioned by the participants, developing an own approach to CSHC takes time, for example to reflect on the aims of CSHC, a physician wants to pursue [39]. Our results on communication strategies suggest that patient-centered approaches may allow physicians to decide individually, which possible aims of CSHC they pursue with which patient.

With regard to content areas, ‘sustainable and healthy lifestyles’ received greatest attention in the interviews. This may seem surprising because other research often focuses on addressing climate change impacts and adaptation [7, 8, 40]. Yet, this might be due to the specific sample including many physicians who are highly engaged in climate action privately. They may be more motivated than the average physician to promote climate change mitigation measures for society. In a cross-sectional population survey, German adults’ mean interest in content areas of CSHC was slightly higher for climate impacts and adaptation than for healthy and sustainable lifestyles [26]. In that survey, participants found it most important that counseling was linked to their personal health, which was also an important factor, discussed by our participants [26].

The “new” content area of climate-sensitive clinical decision-making had been excluded from the CSHC framework, as the studies identified in the review from 2023 did not address communication aspects with patients in the context of clinical decision-making [8]. However, ethical grounding and communication of climate-sensitive clinical decision-making has received increasing scientific attention lately [41–43]. For instance, patients did express interest in climate and sustainability aspects in health care and did not want their doctors to gatekeep this information [41]. Thus, communication of climate-sensitive clinical decisions-making should be considered for inclusion into a revised version of the CSHC framework.

Most communication strategies identified in the CSHC framework, were also mentioned by the participants of this study. A recurring emphasis in the identified health counseling strategies was on patient-centered communication. This aligns well with findings from a qualitative study with patients, which were recruited from a sub-sample of physicians interviewed in this study. Griesel et al. identified patient-centered communication as an important contributor to patients’ acceptance of CSHC [22]. Also other factors contributing to patients’ acceptance, such as a good therapeutic relationship, or factors challenging acceptance, such as fear of politization, resonate well with the physicians’ perspectives on implementation reflected in this study [22].

Although participants readily accepted the study focus on CSHC, they referred to concepts broader than climate change, such as biodiversity, environment, animal welfare, sustainability and planetary health. We consider CSHC is still a valid term for this study because it provides a clear conceptual distinction for academic purposes. Yet, we embrace that participants have a broader view of sustainability and planetary health and also see the concept of CSHC within the broader concept of planetary health. Future research should investigate which terminology allows for conceptual clarity, a holistic approach to planetary health and easy communication with health professionals and patients.

Physicians had cautious expectations with regard to the actual impact of CSHC, but considered the reinforcement of patients’ existing intentions or providing them with food for thought as a realistic short-term goal. This aligns with stages from the transtheoretical model of change (stage of change) [44] and underlines that CSHC might not be an immediate game changer in behavioral climate change mitigation, but could be one small wheel to turn with regard to sustainable transformation of society.

### Research question 2: what supports physicians in conducting CSHC?

In terms of facilitators to implement CSHC, physician factors were addressed in most detail, possibly due to the nature of the interviews with physicians. Physicians’ inner attitude and perceived responsibility for climate change and health issues seemed to be a strong driver for implementing CSHC in this study. Values and personal commitment have been identified as important principles of planetary health [45]. Furthermore, providing values and confidence has also been identified as key concepts in planetary health education for health professionals [46]. Professional identities provide an ethical framework in which physicians work [47]. They start to form in medical school and throughout the later career and are key for patients, the medical professional him-/herself and the social interaction in a professional team [48]. Studies with medical students have found that professional role perceptions with regard to climate change and health are associated with whether they want to learn about climate change and health in their medical studies [49]. Our results support the idea that integrating planetary health values into professional identity formation at medical schools and in continued medical education [5], will help to implement CSHC and other climate-informed health services [46, 50]. With regard to shaping professional identities and perceived responsibility in climate change and health after the medical school, a study by Kotcher et al. (2025) showed that story telling about role models in climate and health can raise health professionals’ perceived responsibility to engage in climate action [51]

Also other physician, patient and relationship factors identified as supportive of the implementation of CSHC in this study could be subject of educational courses and trainings for medical students and physicians. For instance, teaching should include knowledge about the concept of and touchpoints for CSHC as outlined in the framework and RQ 1, and skills supporting patients’ autonomy and individually consider patients’ level of interest and understanding for CSHC. This also resonates well with principles of patient-centered communication, which should be taught in the context of CSHC. As duration of relationship was considered as one facilitator for CSHC, physician-patient relationships holding over a longer period of time seem to be particularly suitable.

On the level of the health system, factors such as the financing of CSHC or the creation of a planetary health care culture [16, 21, 52] seem to be supportive in implementing CSHC, which might be particularly important for a broader range of physicians, who are not as intrinsically motivated as participants in our sample.

### Strength and limitations

To our knowledge, this is the first study giving empirical evidence about how physicians conduct CSHC and what supports them in implementing it. This provides valuable insights from pioneers to advance the conceptualization and implementation of CSHC in research and practice. The qualitative design was suitable to explore physicians’ experiences with CSHC in-depth. Using a hybrid deductive and inductive approach to qualitative analysis was helpful to validate and add nuances to the pre-existing framework of CSHC [8], which was published (2023) after the conception and data collection of this study (2020/2021). As this framework emerged as a part of the wider research project, it seemed to be suitable to validate and expand this framework in the presented study [53].

For the research question on what supports physicians in implementing CSHC, the deductive-inductive analysis approach was performed iteratively. Usually, deductive codes are developed from existing research and applied as propositions from inception of the study [54]. Yet, according to Bingham such propositions can also emerge in the course of the analysis, so that codes driven from existing research can also be integrated throughout the analysis process and be evaluated in the further analysis [54]. We drew on work on factors influencing patient-centered communication to guide our coding scheme after having identified patient-centered communication as a key component of CSHC. Choosing a theory or existing research to guide coding, entails the risk of confirmation bias by pressing themes into pre-existing categories and overlooking important themes emerging from the data. We tried to mitigate that risk by engaging with the theory, thoroughly checking if the initial coding of the raw data was compatible with the theory and ensure reliability of the primary coder by checking the coding of a subset of the raw material by a second coder and team discussions [55]. Our research-question driven approach to analysis allowed us to focus on the research questions in depth, but gave less room to explore emerging themes, like the considerations of the broader planetary health concept and other environmental issues under the umbrella of “climate-sensitive” health counseling.

While qualitative results are never generalizable to a target population, it is important to note that the purposeful sampling conducted in this study carries several limitations towards the transferability of results to a more diverse set of physicians. We purposefully recruited physicians already conducting CSHC and were therefore very likely to be actively engaged in the climate change and health community due to the recruitment procedures. The strength of this approach is that the participants were experts in CSHC and provided information-rich cases to answer our research questions with regard to concepts and facilitators of CSHC. We want to highlight here that we were explicitly asking for what helps to conduct and implement CSHC, not focusing on barriers, which have been described extensively elsewhere [8, 9]. The limitation of this approach is, that especially the results about what supports implementation of CSHC (e.g. inner attitude towards climate change) might be specific to the context of physicians who were pioneers in CSHC. Therefore, transferability of our findings to a more diverse set of physicians might be limited, and external motivators (such as financial reimbursement for CSHC) more important than in this sample.

In addition, while the sample differed with regard to age, sex, medical specialization and work environment, 15 out of 18 participants were general practitioners or internal specialists in primary care, so that results on additional emphases and extensions to the original CSHC framework might be context-specific for general practitioners and internal specialists. Generally, the statements from the three physicians with other specializations fit into the coding scheme. Still, a more nuanced investigation for specific medical fields like gynecology could be beneficial to give more context-specific results, for instance on touchpoints for CSHC like providing information on heat protection to pregnant women [56]. As other health professions had originally been included in the recruitment, but already excluded from the analysis due to majorly differing approaches to CSHC, further research with other health professions is needed to explore potential differences of CSHC in other medical disciplines.

## Conclusions

The practical insights of this study suggest that the framework for CSHC is valid to guide CSHC in primary care in Germany. As sustainable health care is a rapidly evolving field, regular revisions of the framework seem warranted, for instance considering the inclusion of the content area of clinical decision-making. The validation and specifications of the framework for different medical specializations and other health professions are future directions of research. In the context of primary care in Germany, the current framework seems to be sufficiently mature to facilitate the design of implementation studies, with the objective of establishing the effects of CSHC on patient-level outcomes, which are largely missing to date.

With regard to what can support the implementation of CSHC, medical curricula and trainings for physicians seem to be important tools, as they can address several physician factors identified as important in this context. In particular, curricula and trainings should initiate a reflection on attitudes towards climate change and health as part of professional identity formation. Furthermore, such programs should provide conceptual knowledge about CSHC and deliver skills in patient-centered communication. Health-system factors such as reimbursement of CSHC and promoting a planetary health culture at health-system level may further contribute to its implementation.

## Electronic supplementary material

Below is the link to the electronic supplementary material.

Supplementary Material 1

## Data Availability

The coding scheme and key quotes of participants necessary to support the results are included in the manuscript and supplementary material. Access to anonymized versions of the interview transcripts can be granted upon reasonable request to the authors.

## References

[1] Richardson K, Steffen W, Lucht W, Bendtsen J, Cornell SE, Donges JF, et al. Earth beyond six of nine planetary boundaries. Sci Adv. 2023;9(37):eadh2458. 37703365 10.1126/sciadv.adh2458 PMC10499318

[2] Whitmee S, Haines A, Beyrer C, Boltz F, Capon AG, de Souza Dias BF, et al. Safeguarding human health in the Anthropocene epoch: report of the Rockefeller Foundation-Lancet Commission on planetary health. Lancet. 2015;386(10007):1973–2028. 26188744 10.1016/S0140-6736(15)60901-1

[3] Romanello M, Walawender M, Hsu SC, Moskeland A, Palmeiro-Silva Y, Scamman D, et al. The 2024 report of the Lancet Countdown on health and climate change: facing record-breaking threats from delayed action. Lancet. 2024. 10.1016/S0140-6736(24)01822-1 PMC7616816 39488222

[4] WBGU. Healthy living on a healthy planet. Berlin; 2023.

[5] WHO. Operational framework for building climate-resilient and sustainable health systems. World Health Organization. 2023.

[6] Howard C, MacNeill AJ, Hughes F, Alqodmani L, Charlesworth K, de Almeida R, et al. Learning to treat the climate emergency together: social tipping interventions by the health community. Lancet Planet Health. 2023;7(3):e251–64. 36889866 10.1016/S2542-5196(23)00022-0

[7] Kotcher J, Patel L, Wheat S, Philipsborn R, Maibach E. How to communicate about climate change with patients. BMJ (Clin Res Ed). 2024;385:e079831. 10.1136/bmj-2024-079831 38631729

[8] Quitmann C, Griesel S, Nayna Schwerdtle P, Danquah I, Herrmann A. Climate-sensitive health counselling: a scoping review and conceptual framework. Lancet Planet Health. 2023;7(7):e600–10. 37438001 10.1016/S2542-5196(23)00107-9

[9] Redvers N, Wright K, Hartmann-Boyce J, Tonkin-Crine S. Physicians’ views of patient-planetary health co-benefit prescribing: a mixed methods systematic review. Lancet Planet Health. 2023;7(5):e407–17. 37164517 10.1016/S2542-5196(23)00050-5

[10] Lewandowski AA, Sheffield PE, Ahdoot S, Maibach EW. Patients value climate change counseling provided by their pediatrician: the experience in one Wisconsin pediatric clinic. J Clim Chang Health. 2021;4:100053.

[11] Reismann L, Weber A, Leitzmann M, Jochem C. Climate-specific health literacy and medical advice: the potential for health co-benefits and climate change mitigation. An exploratory study. J Clim Chang Health. 2021;4.

[12] Kotcher J, Maibach E, Miller J, Campbell E, Alqodmani L, Maiero M, et al. Views of health professionals on climate change and health: a multinational survey study. Lancet Planet Health. 2021;5(5):e316–23. 33838130 10.1016/S2542-5196(21)00053-X PMC8099728

[13] Sarfaty M, Bloodhart B, Ewart G, Thurston GD, Balmes JR, Guidotti TL, et al. American Thoracic Society member survey on climate change and health. Ann Am Thorac Soc. 2015;12(2):274–78. 25535822 10.1513/AnnalsATS.201410-460BC PMC5466202

[14] Hathaway J, Maibach EW. Health implications of climate change: a review of the literature about the perception of the public and health professionals. Curr Environ Health Rep. 2018;5:197–204. 29423661 10.1007/s40572-018-0190-3 PMC5876339

[15] Mezger NCS, Thöne M, Wellstein I, Schneider F, Litke N, Führer AG, et al. Climate protection in practices - current status, motivation and challenges in outpatient care. Z Evid Fortbild Qual Gesundhwes. 2021;166:44–54. 34656461 10.1016/j.zefq.2021.08.009

[16] André H, Gonzalez Holguera J, Depoux A, Pasquier J, Haller DM, Rodondi P-Y, et al. Talking about climate change and environmental degradation with patients in primary care: a cross-sectional survey on knowledge, potential domains of action and points of view of general practitioners. Int J Environ Res Public Health. 2022;19(8):4901. 35457768 10.3390/ijerph19084901 PMC9029888

[17] Campbell E, Uppalapati S, Kotcher JE, Thier K, Ansah P, Gour N, et al. Activating health professionals as climate change and health communicators and advocates: a review. Environ Res: Health. 2025.

[18] eG GQuE. Klimasensible Versorgung: Gesundheit und Klima im Fokus ärztlicher Praxis Nürnberg: Gesundheitsnetz Qualität und Effizienz eG. 2025 Available from: https://www.gesundheitsnetznuernberg.de/gesundheit-und-klima-klimasensible-versorgung/.

[19] Acadamy PH. Transformative Communication 1: climate-sensitive health counselling Berlin: Deutsche Allianz Klimawandel und Gesundheit (KLUG) e.V. 2022. Available from: https://planetary-health-academy.de/transformative-kommunikation-1-klimasensible-gesundheitsberatung/.

[20] H-Uh B-W. Klimaresiliente Versorgung Stuttgart: Hausärztinnen- und Hausärzteverband Baden-Württemberg e. V. 2025. Available from: https://www.haevbw.de/klima-versorgung.

[21] Redvers N, Hartmann-Boyce J, Tonkin-Crine S. Patient-planetary health co-benefit prescribing in a circumpolar health region: a qualitative study of physician voices from the Northwest Territories, Canada. BMJ Open. 2024;14(3):e081156. 38431297 10.1136/bmjopen-2023-081156 PMC10910660

[22] Griesel S, Schwerdtle PN, Quitmann C, Danquah I, Herrmann A. Patients’ perceptions of climate-sensitive health counselling in primary care: qualitative results from Germany. Eur J Gen Pract. 2023;29(1):2284261. 38010828 10.1080/13814788.2023.2284261 PMC10773651

[23] Herrmann A, Krippl N, Fischer H, Nieder J, Griesel S, Bärnighausen T, et al. Acceptability of health-only versus climate-and-health framings in lifestyle-related climate-sensitive health counselling: results of a randomised survey experiment in Germany. Lancet Planet Health. 2025;9(6):e456–66. 40516537 10.1016/S2542-5196(25)00110-X

[24] den Boer Acl, Teherani A, de Hoop E. Discussing climate change and other forms of global environmental change during the clinical encounter: exploring us physicians’ perspectives. J Clim Chang Health. 2021;4:100058.

[25] Meuser M, Nagel U. Das Experteninterview — konzeptionelle Grundlagen und methodische Anlage. In: Pickel S, Pickel G, Lauth H-J, Jahn D, editors. Methoden der vergleichenden Politik- und Sozialwissenschaft: VS Verlag für Sozialwissenschaften. 2009. p. 465–79.

[26] Krippl N, Mezger N, Danquah I, Nieder J, Griesel S, Schildmann J, et al. Climate-sensitive health counselling in Germany: a cross-sectional study about previous participation and preferences in the general public. BMC Public Health. 2024;24(1):1–11. 38844875 10.1186/s12889-024-18998-6 PMC11155184

[27] Chaudoir SR, Dugan AG, Barr CH. Measuring factors affecting implementation of health innovations: a systematic review of structural, organizational, provider, patient, and innovation level measures. Implementation Sci: IS. 2013;8:22. 23414420 10.1186/1748-5908-8-22 PMC3598720

[28] Damschroder LJ, Aron DC, Keith RE, Kirsh SR, Alexander JA, Lowery JC. Fostering implementation of health services research findings into practice: a consolidated framework for advancing implementation science. Implementation Sci: IS. 2009;4:50. 19664226 10.1186/1748-5908-4-50 PMC2736161

[29] Quitmann C, Sauerborn R, Danquah I, Herrmann A. Reducing the carbon footprint of a German university hospital: perspectives from hospital stakeholders. J Clim Chang Health. 2023;12:100247.

[30] Tong A, Sainsbury P, Craig J. Consolidated criteria for reporting qualitative research (coreq): a 32-item checklist for interviews and focus groups. Int J Qual Health Care. 2007;19(6):349–57. 17872937 10.1093/intqhc/mzm042

[31] Patton MQ. Qualitative evaluation and research methods. SAGE Publications, inc; 1990.

[32] Krolewski R. Klimaschutz und Gesundheit: Die Patienten informieren. Deutsches Ärzteblatt. 2022;119(10).

[33] Herrmann A, Krolewski R. Gesundheitsberatung im Kontext von Planetary Health. In: Traidl-Hoffmann C, Schulz C, Herrmann M, Simon B, editors. Planetary Health: Klima, Umwelt und Gesundheit im Anthropozän. MWV; 2021.

[34] Morse JM. Data were saturated. Los Angeles, CA: Sage Publications Sage CA; 2015. p. 587–88.

[35] Gläser J, Laudel G. 4.3 Konstruktion des Inteviewleitfadens. In: Gläser J, Laudel G, editors. Experteninterviews und qualitative inhaltsanalyse. Wiesbaden: VS Verlag; 2006. p. 142–50.

[36] Archibald MM, Ambagtsheer RC, Casey MG, Lawless M. Using zoom videoconferencing for qualitative data collection: perceptions and experiences of researchers and participants. Int J Qual. 2019;18:1609406919874596.

[37] Kuckartz U. Qualitative Inhaltsanalyse: Methoden, Praxis, Computerunterstützung (3., überarbeitete Auflage). Grundlagentexte Methoden. Weinheim Basel: Beltz Juventa; 2016.

[38] Epstein RM, Franks P, Fiscella K, Shields CG, Meldrum SC, Kravitz RL, et al. Measuring patient-centered communication in patient–physician consultations: theoretical and practical issues. Soc Sci Med. 2005;61(7):1516–28. 16005784 10.1016/j.socscimed.2005.02.001

[39] Herrmann A, Mews C, Hansen H, Lenzer B, Schwienhorst-Stich E-M, Quitmann C. Klimasensible Gesundheitsberatung. Z für Allgemeinmedizin. 2023;99(8):426–36.

[40] Senay E, Sarfaty M, Rice MB. Strategies for clinical discussions about climate change. Ann Intern Med. 2021;174(3):417±. 33315471 10.7326/M20-6443 PMC7737930

[41] Hantel A, Senay E, Richie C, Revette A, Nava-Coulter B, Hlubocky FJ, et al. A focus group study of ethical issues during climate-informed health decision-making. Nat Clim Chang. 2024.

[42] Müller F, Skok JI, Arnetz JE, Bouthillier MJ, Holman HT. Primary care clinicians’ attitude, knowledge, and willingness to address climate change in shared decision-making. J Am Board Fam Med. 2024;37(1):25–34. 37385719 10.3122/jabfm.2023.230027R1

[43] Kuiter SG, Herrmann A, Mertz M, Quitmann C, Salloch S. Should healthcare professionals include aspects of environmental sustainability in clinical decision-making? A systematic review of reasons. BMC Med Ethics. 2025;26(1):78. 40611029 10.1186/s12910-025-01230-4 PMC12226885

[44] Petrocelli JV. Processes and stages of change: counseling with the transtheoretical model of change. J Couns Dev. 2002;80(1):22–30.

[45] Prescott SL, Logan AC, Albrecht G, Campbell DE, Crane J, Cunsolo A, et al. The canmore declaration: statement of principles for planetary health. Challenges. 2018;9(2):31.

[46] Shaw E, Walpole S, McLean M, Alvarez-Nieto C, Barna S, Bazin K, et al. Amee consensus statement: planetary health and education for sustainable healthcare. Med Teach. 2021;43(3):272–86. 33602043 10.1080/0142159X.2020.1860207

[47] Monrouxe LV. Theoretical insights into the nature and nurture of professional identities. Teach Med Professionalism: Supporting Devel Prof Identity. 2016;2:37–53.

[48] Rees CE, Monrouxe LV. Who are you and who do you want to be? Key considerations in developing professional identities in medicine. Med J Aust. 2018;209(5):202–03. 30157410 10.5694/mja18.00118

[49] Rybol L, Nieder J, Amelung D, Hachad H, Sauerborn R, Depoux A, et al. Integrating climate change and health topics into the medical curriculum–a quantitative needs assessment of medical students at Heidelberg University in Germany. GMS J Med Educ. 2023;40(3). 10.3205/zma001618 PMC10291352 37377571

[50] Barna S, Maric F, Simons J, Kumar S, Blankestijn PJ. Education for the Anthropocene: planetary health, sustainable health care, and the health workforce. Med Teach. 2020;42(10):1091–96. 32805141 10.1080/0142159X.2020.1798914

[51] Kotcher J, Badullovich N, Ahmed M, De Alwis D, Maibach EW. Role model stories can increase health professionals’ interest and perceived responsibility to engage in climate and sustainability actions. J Clim Chang Health. 2024;18:100291. 10.1016/j.joclim.2023.100291 PMC12851224 41646520

[52] MacNeill AJ, McGain F, Sherman JD. Planetary health care: a framework for sustainable health systems. Lancet Planet Health. 2021;5(2):e66–8. 33581064 10.1016/S2542-5196(21)00005-X

[53] Fife ST, Gossner JD. Deductive qualitative analysis: evaluating, expanding, and refining theory. Int J Qual. 2024;23:16094069241244856.

[54] Bingham AJ. From data management to actionable findings: a five-phase process of qualitative data analysis. Int J Qual. 2023;22:16094069231183620.

[55] Proudfoot K. Inductive/Deductive hybrid thematic analysis in mixed methods research. J Mix Methods Res. 2023;17(3):308–26.

[56] Lehmann S, Willems L, Mezger NCS. Klimasensible Gesundheitsberatung in der Frauenheilkunde. Die Gynäkologie. 2025;1–8.

