# Supplementary material for: Implementing climate-sensitive health counselling: a qualitative study with physicians in Germany
Source: BMC Health Serv Res. 2025 Dec 1;25:1557. doi: 10.1186/s12913-025-13701-w (PMC12670745; doi:10.1186/s12913-025-13701-w)
Supplement: Supplementary file 1 — Supplementary Material 1 [file 12913_2025_13701_MOESM1_ESM.docx]

# Appendix

Manuscript: Implementing Climate-Sensitive Health Counselling: A qualitative Study with Physicians in Germany

## Interview Guide (translated version, original version was in German)

### Explanation of the structure of the guide with the main topic and structure of a question group

| **A. (letter) Main topic of a block** |
| --- |
| Information/introductory sentence for one or more main questions |
| 1. **(numeration): Top question (will be raised)**    - Sub-questions:    - **In bold: question will be raised in any case**    - Normally printed: Question is only raised if the topic has not yet been addressed by the study participant and there is enough time available      - Internal notes |
| Comments for one or more main questions   - Explanatory summary of the question group |

### Interview Guide

| **Explanation for study participants** |
| --- |
| **1. Welcome and introduction** |
| 1. **Renewed naming of**    - Topic: Climate change and health in general practitioner counselling    - Aim of the investigation:      1. Conception of a "climate consultation hour", i.e. answering the question of how doctors can integrate topics on climate change and health into (general) medical consultations      2. Development of a pragmatic guideline for the doctor-patient consultation |
| 1. **Renewed references to ethical aspects**    - Tape recording    - Data protection    - Willingness and option of cancellation of the declaration of consent |
| 1. **Notes on the interview process:**    - Duration approx. 1 hour    - Guide contains topics to be covered in the interview, but also leaves room for unforeseen content.    - Mainly open questions, follow-up questions possible at any time.    - The interview is not a knowledge test. There is no "right" and "wrong", it is about examining YOUR judgements, perceptions and thoughts. We want to learn from you.    - Questions (including dichotomous questions) should be   understood as narrative stimuli  x Interviewer: start audio recording x |
| **Socio-economic data** |
| 1. **Age:** 2. **Gender:** 3. **State, city:** 4. **Specialist training and additional qualifications:** 5. **Practicing doctor since:** 6. **Practice type:** |
| 1. **Warm-up questions on climate change and health** |
| 1. **Before we start with the actual topic ‘Climate consultation hour’, I would like to ask you a few questions about climate change and health?** 2. **We have chosen you for the study because we know that you have already dealt with the topic of climate change in one way or another. How did you become aware of this topic?** 3. **What is your motivation to deal with the topic of climate change and health?** 4. **Which topics in the field of climate change and health do you find particularly relevant to your work as a doctor?** |
| **B. Climate consultation - experiences to date** |
| 1. **This study is entitled ‘Climate Consultation Hour’. What do you associate with this buzzword?**  - Have you already heard of this concept somewhere? - If so, in what context? What do you associate with it? - If not, please continue.  1. **A provisional working definition of a climate consultation is the targeted inclusion of topics relating to climate change and health in the doctor-patient consultation.**  - What do you think about this?  1. **Have you already had any experience of incorporating aspects of climate change and health into your own medical practice? If so,**  - What was it like for you personally to talk about these issues with your patients? - What did you find easy? - What did you find difficult?  1. **How did your patients react?** |
| **C. Goals of the climate consultation** |
| 1. **What goals do you pursue (would you like to pursue) when discussing climate change in doctor-patient consultations?** 2. **To what extent do you think it is appropriate as a doctor to also pursue socio-political goals in the doctor-patient consultation?** |
| **D. Topics** |
| 1. **What specific topics in the area of climate change and health have you addressed with your patients?**  - Why did you address these topics in particular?  1. **Which topics do you find particularly important?** 2. **Which topics do you find particularly difficult to address? Why?** 3. **Now I will mention a few topics that we have not yet addressed. What**   **What do you think of these topics as the content of a climate consultation?**  Explicitly address the following topics that have not yet been mentioned:   - Effects of climate change on health   Adaptation to climate change   - Lifestyle and co-benefits of climate protection - other planetary health topics e.g. plastic use |
| **E. Target groups** |
| 1. **In your opinion, which patients are particularly open to climate consultation topics?** 2. **Which patients are particularly difficult to talk to about these topics?** 3. **Which patients would you find it particularly important to talk to about climate change and health issues?**   **18. Which patients do you think are particularly affected by climate change?** |
| **F. Methods/occasions** |
| **19. In which situation in your consultation do you discuss climate change with your patients (or can you imagine discussing climate change with your patients)?**  **20. What type of dialogue do you choose (can you imagine choosing) when discussing such topics with a patient?**   - Why this particular type of conversation? - Which types of counselling do you generally prefer in your consultations?   **21. When do you generally conduct consultations on health-promoting behaviour and lifestyles?**   - How often do you do this? - To what extent do you feel that this counselling actually leads to your patients changing their behaviour? - What do you find particularly difficult? What helps you with successful counselling?   **22. Have you ever tried to incorporate aspects of climate protection into lifestyle counselling?**   - **If yes, how have you done this?** - **If not, what has prevented you from doing so?** - To what extent is it a goal of your counselling to explicitly address such topics? |
| **G. Skills, attitudes** |
| **23. What basic attitude do you have towards your patients in conversations?**   - **To what extent do you find this basic attitude in the area of ‘climate consultations’ helpful**   **or a hindrance?**  **24. Have you ever heard of the concept of Planetary Health?**   - **If yes, what does this concept mean for your work as a general health counselor?** - **If no: Planetary health is defined as the health of people and the social and natural**   **systems on which it is based. The Planetary Health movement therefore sees the preservation of natural systems as fundamental to the preservation of human health.**   - **What do you think of this concept?** - **o What does this mean for your medical practice?** |
| **H. Conclusion of the conversation** |
| **25. Looking back on our conversation: What particularly caught your attention?**  **26. Are there still things that you find important that we have not yet addressed?** |

## Coding Scheme Research Question 1: How do physicians conduct CSHC?

This coding scheme was based on the four categories of the conceptual framework of climate-sensitive health counselling (1). All categories were applied deductively from the framework (2). At the level of sub-categories we indicate, which subcategories were applied deductively and which ones were developed inductively from the data.

| **Cate- gory** | **Sub-category** | **Second-level subcategory (marked with an*) and Codes** |
| --- | --- | --- |
| **Aims (deductive)** | Protect and promote individual and public health (deductive) | - Patients health - Public health - Planetary health |
|  | Encourage Climate action and lifestyle change (deductive) | - Emphasizing health and well-being as motivation for lifestyle change - Emphasizing impact on societal processes - Strengthening patients’ self-efficacy (2^nd^ level sub-category)* |
|  | Increase climate change and health knowledge and awareness (deductive) | - Climate change and health nexus - Climate crisis - Climate justice - Being part of nature - Air pollution |
|  |  |  |
| **Conten Areas (deductive)** | Health impacts and adaptation (deductive) | - Climate-sensitive diseases and vulnerabilities (2^nd^ level sub-category)* - Climate-sensitive phenomena (2^nd^ level sub-category)* |
|  | Sustainable and healthy lifestyles (deductive) | - Dietary behavior - Active Mobility - Connection to nature and mindfulness - Other consumption behaviour |
|  | Climate action and policies (deductive) | - Opportunities to engage in climate action - Role of politics |
|  | Clinical decision making (inductive) | - Overdiagnosis and overtherapy - Environmentally friendly medication - Natural therapies (often in the German term *“Naturheilkunde”* = naturopathy) |
| **Communication Strategies (deductive)** | Climate change communication strategies (deductive) | - Climate and health co-benefits - Being a role model and acting authentically |
|  | Health counselling strategies (deductive) | - Patient-centered approaches to communication (2^nd^ level sub-category)** - shared decision making - active listening - motivational interviewing - strength-based approach - narrative approach |
|  |  |  |
| **Integration into routine cealth are (deductive, wording adapted)** | Routine health care activities (deductive) | - check-ups - results of medical examinations like blood tests - regular lifestyle counselling - vaccinations - disease management programs - home visits - skin cancer screening |
|  | Symptoms, diagnoses, or social issues (inductive) | - obesity - diabetes - cardiovascular diseases - mental health issues - musculoskeletal issues/pain - stress - asthma - breastfeeding - pregnancy - cancer - erectile dysfunction - intestinal polyp - renal disease - metabolic issues - diverticulitis - attack of gout - job situation |

* Details within the categories are named in the research paper to give a more concrete understanding of these 2^nd^ level sub-categories. / ** Patient-centered approaches to communication were coded more extensively as a second level of sub-category. Details are shown in Table 3 of the research paper.

## Coding Scheme Research Question 2: What helps physicians conduct CSHC?

This coding scheme was based on a paper describing factors influencing implementation of patient-centered communication by Epstein et al 2005 (3). Categories are deductively derived from the framework of this paper. Sub-categories are mainly derived inductively from our interviews, but partly inspired by reflections of subcategories from Epstein et al.

| **Cate- gory** | **Sub-category** | **Codes** |
| --- | --- | --- |
| **Physician factors (deductive)** | Development of an inner attitude | - Personal motivation regarding climate change and health - Development of personal motivation regarding climate change and health - Attitude regarding climate change and health - Perceived responsibility for CSHC |
|  | Experienced patient response | - Experienced patient response - Perceived impact on patients |
|  | Assessing appropriateness of CSHC | - Reasons for appropriateness - Reasons why and what is inappropriate |
|  | Concepts of and approach to CSHC | - Own approach to CSHC - Defined concept of CSHC |
|  | Awareness of touchpoints for CSHC | - Based on specific symptoms or diagnoses - Based on routine healthcare activities - Every patient is screened for opportunities – always having CSHC in mind |
|  | Supporting patient autonomy (inspired by Epstein et al.) | - Not being authoritarian, paternalistic or intrusive |
|  | Knowing the patient’s bio-psycho-social circumstances (inspired by Epstein et al.) | - Knowing the patient’s bio-psycho-social circumstances |
| **Patient factors (deductive)** | (Individual) health in the focus of what is important for patients | - (Individual) health in the focus of what is important for patients |
|  | Level of interest and openness of patients | - Groups with existing interest and openness - Groups with lacking interest and openness - Some patients actively address topics of CSHC - Some patients have limited opportunities to lifestyle changes |
|  | Level of understanding | - Broader context is too far away of patients’ reality - Broader context is too complex for certain patients |
|  | Severity of illness (inspired by Epstein et al.) | - Severity of illness |
| **Health systems factors (deductive)** | Culture in the practice team | - Enabling culture in practice team - Missing support and interest of practice team |
|  | Integrating planetary health into education of physicians | - Training or ‘Zusatzbezeichung’ - Lacking training opportunities with regard to climate change and health |
|  | Reimbursement system | - Reimbursement of CSHC (also as a political signal) - CSHC is currently not reimbursed |
|  | Visit length and frequency (inspired by Epstein et al.) | - Insufficient time - Sufficient time - Regularly seeing patients |
| **Relationship factors (deductive)** | Duration of relationship (inspired by Epstein et al.) | - Duration of relationship |
|  | Shared values and beliefs (inspired by Epstein et al.) | - Shared values and beliefs |
|  | Trust (inspired by Epstein et al.) | - Trust |

## Consolidated criteria for reporting qualitative studies (COREQ): 32-item checklist

Based on: Tong A, Sainsbury P, Craig J. Consolidated criteria for reporting qualitative research (COREQ): a 32-item checklist for interviews and focus groups. *International Journal for Quality in Health Care*. 2007. Volume 19, Number 6: pp. 349 – 357

| **Number of COREQ-Item** | **Guiding questions/ description** | **Reported on Page #** |
| --- | --- | --- |
| **Domain 1: Research team and reﬂexivity** |  |  |
| *Personal Characteristics* |  |  |
| 1. Interviewer/facilitator | Which author/s conducted the interview or focus group? | Page 7 |
| 2. Credentials | What were the researcher’s credentials? E.g. PhD, MD | Page 7 |
| 3. Occupation | What was their occupation at the time of the study? | Page 7 |
| 4. Gender | Was the researcher male or female? | Page 1 |
| 5. Experience and training | What experience or training did the researcher have? | Page 7 |
| *Relationship with participants* |  |  |
| 6. Relationship established | Was a relationship established prior to study commencement? | Page 7  . |
| 7. Participant knowledge of the interviewer | What did the participants know about the researcher? e.g. personal goals, reasons for doing the research | Page 7 |
| 8. Interviewer characteristics | What characteristics were reported about the inter viewer/facilitator? e.g. Bias, assumptions, reasons and interests in the research topic | Page 7 and 9 |

…continued on next page.

| **Domain 2: study design** |  |  |
| --- | --- | --- |
| *Theoretical framework* |  |  |
| 9. Methodological orientation and Theory | What methodological orientation was stated to underpin the study? e.g. grounded theory, discourse analysis, ethnography, phenomenology, content analysis | Page 5,6,7,9 |
| *Participant selection* |  |  |
| 10. Sampling | How were participants selected? e.g. purposive, convenience, consecutive, snowball | Page 6 |
| 11. Method of approach | How were participants approached? e.g. face-to-face, telephone, mail, email | Page 6,7 |
| 12. Sample size | How many participants were in the study? | Page 10 |
| 13. Non-participation | How many people refused to participate or dropped out? Reasons? | Page 6 |
| *Setting* |  |  |
| 14. Setting of data collection | Where was the data collected? e.g. home, clinic, workplace | Page 7  . |
| 15. Presence of non-participants | Was anyone else present besides the participants and researchers? | Page 6 |
| 16. Description of sample | What are the important characteristics of the sample? e.g. demographic data, date | Page 10 |
| *Data collection* |  |  |
| 17. Interview guide | Were questions, prompts, guides provided by the authors? Was it pilot tested? | Page 6,7 and Appendix 1 |
| 18. Repeat interviews | Were repeat inter views carried out? If yes, how many? | Page 7 |
| 19. Audio/visual recording | Did the research use audio or visual recording to collect the data? | Page 7 |
| 20. Field notes | Were ﬁeld notes made during and/or after the interview or focus group? | Page 7 |
| 21. Duration | What was the duration of the interviews or focus group? | Page 10 |
| 22. Data saturation | Was data saturation discussed? | Page 6 |
| 23. Transcripts returned | Were transcripts returned to participants for comment and/or correction? | Page 8 |
| **Domain 3: analysis and ﬁndings** |  |  |
| *Data analysis* |  |  |
| 24. Number of data coders | How many data coders coded the data? | Page 8 |
| 25. Description of the coding tree | Did authors provide a description of the coding tree? | Appendix 2 and 3 |
| 26. Derivation of themes | Were themes identiﬁed in advance or derived from the data? | Page 7,8 |
| 27. Software | What software, if applicable, was used to manage the data? | Page 7 |
| 28. Participant checking | Did participants provide feedback on the ﬁndings? | Page 8 |
| *Reporting* |  |  |
| 29. Quotations presented | Were participant quotations presented to illustrate the themes/ﬁndings? Was each quotation identiﬁed? e.g. participant number | Page 11-23 |
| 30. Data and ﬁndings consistent | Was there consistency between the data presented and the ﬁndings? | Page 11-23 |
| 31. Clarity of major themes | Were major themes clearly presented in the ﬁndings? | From page 11-23 |
| 32. Clarity of minor themes | Is there a description of diverse cases or discussion of minor themes? | From page 11-23 |

## References

1. Quitmann C, Griesel S, Nayna Schwerdtle P, Danquah I, Herrmann A. Climate-sensitive health counselling: a scoping review and conceptual framework. The Lancet Planetary Health. 2023;7(7):e600-e10.

2. Quitmann C, Griesel S, Nayna Schwerdtle P, Danquah I, Herrmann A. Climate-sensitive health counselling: a scoping review and conceptual framework. The Lancet Planetary Health. 2023;7(7).

3. Epstein RM, Franks P, Fiscella K, Shields CG, Meldrum SC, Kravitz RL, et al. Measuring patient-centered communication in patient–physician consultations: theoretical and practical issues. Social science & medicine. 2005;61(7):1516-28.
